# A Bayesian Reconstruction of a Historical Population in Finland, 1647–1850

**DOI:** 10.1007/s13524-020-00889-1

**Published:** 2020-06-09

**Authors:** Miikka Voutilainen, Jouni Helske, Harri Högmander

**Affiliations:** 1 Department of History and Ethnology, University of Jyvaskyla, Jyvaskyla, Finland; 2 Department of Mathematics and Statistics, University of Jyvaskyla, Jyvaskyla, Finland; 3 Department of Science and Technology, Linköping University, Campus Norrköping, Norrköping, Sweden

**Keywords:** Population history, Population growth, Early modern era, Bayesian estimation

## Abstract

This article provides a novel method for estimating historical population development. We review the previous literature on historical population time-series estimates and propose a general outline to address the well-known methodological problems. We use a Bayesian hierarchical time-series model that allows us to integrate the parish-level data set and prior population information in a coherent manner. The procedure provides us with model-based posterior intervals for the final population estimates. We demonstrate its applicability by estimating the long-term development of Finland’s population from 1647 onward and simultaneously place the country among the very few to have an annual population series of such length available.

**Electronic supplementary material:**

The online version of this article (10.1007/s13524-020-00889-1) contains supplementary material, which is available to authorized users.

## Introduction

Generalizations about historical human population development (e.g., Kremer 1993; Livi-Bacci 2017; Maddison 2001; Madsen et al. 2019; McEvedy and Jones 1978) are complicated by a lack of available time series. The further back we go, the more prominent the issue becomes; only a few countries have annual population series of sufficient quality dating back to the early 1800s, let alone the early modern era and beyond.

The most impressive and well-known of the historical population series available is Wrigley and Schofield’s (1981) population history of England, which dates as far back as 1541. Of the few other annual time series available, Portugal has one dating back to the early 1500s (Palma et al. 2017); northern Italy, Sweden, and Germany have series that reach the mid-1600s (Edvinsson 2015; Galloway 1994; Palm 2001; Pfister and Fertig 2010); Norway, Iceland, and France have series from the 1700s onward (Drake 1969; Henry and Blayo 1975; *Tölfræðihandbók*^1984^); and various other countries have at least local demographic time series that shed light on population development during the seventeenth and eighteenth centuries (see, e.g., Durand 1977; Glass and Eversley 1965; Jannetta and Preston 1991; Jutikkala 1965; Langlois 1935).

The scarcity of long-term population series is a major problem because these are vital inputs for many fields of history, demography, and economics. Not only do they provide an essential contextual macro-economic variable in their own right (Edvinsson 2015; Palma et al. 2017), but they are also of substantial analytical interest. Developments in the literature on long-term economic growth (e.g., Galor and Weil 2000; Strulik and Weisdorf 2014) have sparked growing interest in pre-industrial population dynamics (e.g., Chiarini 2010; Madsen et al. 2019; Møller and Sharp 2014; Nicolini 2007). This strand of research has scrutinized whether the stagnant levels of pre-industrial income per capita were due to demographic responses to variations in living standards and whether demographic fluctuations hold the key to understanding how pre-industrial poverty was eventually overcome (Lagerlöf 2003; Voigtländer and Voth 2013). More recently, historical population trajectories have proven useful in quantifying future demographic developments and providing points of comparison how population trends shape economic growth over longer periods (e.g., Cervellati et al. 2017).

To facilitate future research on long-term population growth, we contribute to the literature in three respects. First, we provide a novel methodology for estimating macro-level population development from scattered and (especially in early periods) low-quality microdata. The methodology integrates parish records with existing literature by incorporating the latter as prior information, which allows us to create a coherent aggregate of annual demographic events and existing population (census) benchmarks.

Second, as a pioneering study, our research provides model-based uncertainty (i.e., posterior) intervals for our population estimates. This is of high importance because perhaps the most crucial omission in historical population reconstructions thus far has been unavailable uncertainty estimates, leaving the door open for conflicting interpretations of respective population developments.

Third, by applying the proposed method, we provide a historical reconstruction of Finnish population beginning from the mid-1600s, which places Finland among the few countries that have such a long annual population series available. This length will allow future research to use modern growth theories to investigate Finnish economic development, which will add to our understanding of the pre-industrial movements of a population living not only at the political and economic peripheries of the early modern world but also at the northern limits of agricultural viability (e.g., Holopainen and Helama 2009).

## Historical Population Estimates

Population censuses are often available from the nineteenth century onward. To produce long-term time series from periods before censuses started, researchers have generally relied on two principal sources of information: fiscal records and ecclesiastical registers.

*Fiscal records* are related to tax payments (see, e.g., Broadberry et al. 2015; Jutikkala 1957; Palm 2000; Palma et al. 2017), and although they provide a cross-sectional (and occasionally annual) look at the taxpaying population, they are not generally regarded as a good substitute for censuses. Wrigley and Schofield (1981:33–34), for instance, decided to ignore all surveys of a fiscal nature in their English population history, “not only because they were notoriously prone to evasion, but also because it is impossible to specify at all accurately the proportion of the population that was legally exempt” (for a more general discussion, see Jütte 1996). Because of this notoriety, historians have typically resorted to more accurately collected special taxes (such as the Swedish *Älvsborgs lösen* of 1571 and the 1610s; see, e.g., Palm 2000, 2001) instead of annually collected taxes, which are often perceived as deteriorating in quality over time as a result of tax evasion. High-quality one-off taxes might be available only once or twice over considerable periods, which means that tax records, at their best, tend to provide only benchmark cross-sections of a population. Interpolation between benchmarks (e.g., Krantz 2017; van Zanden and van Leeuwen 2012; see also Bassino et al. 2019; Madsen et al. 2019) not only provides a rather simple picture of the respective population history but also risks creating a misleading one, wholly dependent on the specific year used for the benchmarks.^1^

When and where they are available, *parish registers* (baptisms, burials, and marriages) have thus formed the staple source for demographic history. To obtain macro-level estimates from these, parish (or other micro-unit) data are aggregated by using fixed-effects regression models (e.g., Pfister and Fertig 2010, see also Clark 2007), inverse weights (e.g., Eriksson et al. 2008), or index series (Galloway 1994; Palm 2001). The resulting aggregate series are then used to make projections from known population benchmarks (e.g., Galloway 1994; Palm 2001; Palma et al. 2017; Wrigley and Schofield 1981).

Undeniably, the best known of the historical population estimates that rely on parish data is the English population history by Wrigley and Schofield (1981). Notwithstanding the folklore (e.g., Lee 1985; Lindert 1983; Møller and Sharp 2014; Razzell 1993; Ruggles 1999) surrounding the possible pitfalls of the series, this landmark study (and its subsequent recalculations from family reconstitutions) has gained wide recognition. Broadberry et al. (2015:4), for example, stated that “[a]ggregate development of England’s population since 1541 is now firmly established, and there is little disagreement respecting the population of the rest of Great Britain after 1700.”

As data quality and availability deteriorate, the interpretations start to differ. English population development before the mid-1500s is more heavily contested. The largest disagreement surrounds the peak in population before the Black Death (e.g., Broadberry et al. 2015; Clark 2007; Postan 1966; Russell 1948); some estimates make the population as low as 3.7 million, whereas others put it as high as 6 million. Similar large differences remain, for instance, between the pre-1650s Italian (Alfani 2007; Cipolla 1965; Malanima 2003) and Swedish (Edvinsson 2015; Palm 2000, 2001) population estimates. Lennart Anderson Palm’s (2000, 2001) original Swedish estimates for 1571 and 1620 suggested populations of approximately 650,000 and 850,000, respectively. Edvinsson (2015), on the other hand, has argued for a population of between 800,000 and 900,000 in 1571 and of roughly 1.07 million in 1620—a proposition most recently accepted by, among others, Schön and Krantz (2015) and criticized by Palm (2016).

These kinds of disagreements highlight the fact that reconstituting historical populations is not an exact science—the estimated path has to stand firm against countless equally believable ones (see, e.g., Lee 1985).^2^ Thus, when adjusting Palm’s (2001) figures, Edvinsson (2015:169) acknowledged that a “[c]ertain amount of guesswork is necessary”; Broadberry et al. (2015:22) estimated the medieval population with “significant margins of error”; van Zanden and van Leeuwen (2012) provided a “very tentative” estimate of the historical Dutch population; Cipolla (1965:572) suggested a wide confidence interval for his Italian estimates (±15%); and even the esteemed Wrigley and Schofield (1981:152) described their own estimate as “tolerably reliable.”^3^

The reasons for these reservations are well known. Historical parish data rarely qualify “fresh out of the box”; a mere aggregation (regardless of the method used) will rarely produce a reliable population series. Parish registers tend to have various defects that can lead to implausible (even negative) population levels when back-projections are made by adding deaths and subtracting births from a benchmark estimate. These defects fall under roughly four headings.

First, baptisms are not the same as births, nor are burials equal to deaths. Historical parish registers were not compiled so much to track population movements as to track the number of taxpaying parishioners. Hence, for example, stillbirths and infant deaths often went unrecorded (see, e.g., Pitkänen and Laakso 1999; Wrigley 1997). Second, early modern ecclesiastical registers were kept somewhat unevenly, and central authorities were unable to enforce the record-keeping practice uniformly. Some parishes began recording vital events earlier than others with interruptions in the process. Problems associated with this increases the further back in time we go: usually the earliest points in population reconstitutions are based on just a handful of parishes. Third, records are subject to various structural and idiosyncratic omissions. The absence of nonconformists is a well-known problem; fires, wars, and demographic crises and discontinuities in the clerical appointments may have resulted in gaps of various length in the records. Fourth, spatial differences in the long-term population growth patterns and uneven targeting of population crises can result in a biased parish sample. In addition to the well-known problem (Wrigley and Schofield 1981) of varying local growth rates, Pfister and Fertig (2010:26–27) noted that an increasing number of parishes in the sample over time may yield a spurious decline in year-to-year population volatility. Studies covering famines and disease outbreaks (e.g., Alfani 2010:35–38; Meng et al. 2015; Ó Gráda 2009) have shown that regional mortality distribution during a population crisis can vary dramatically from normal background patterns. This can create problems in situations where population development during mortality crises is estimated using data from only a few sample parishes: the English mid-1500s is an apt example, as Møller and Sharp (2014) noted.

Previous literature has relied on two broad measures to address these deficiencies: either correcting the original data or correcting aggregated first estimates. The former method often means that the parish series are simply multiplied by some corrective constant. Finnish scholars have, for instance, routinely increased baptisms by 10% to account for missing births (e.g., Jutikkala 1944; Muroma 1991; see also Wrigley 1977).^4^ However, much more elaborate methods have also been suggested, especially the procedure laid out in Wrigley and Schofield (1981). A well-known correction method is the one Bourgeois-Pichat (1951) used to correct for underregistered (infant) mortality, later applied by, for example, Henry and Blayo (1975), Wrigley (1977), and Pitkänen and Laakso (1999).

Correcting aggregated first estimates usually relies on the assumption that demographic series display contextually independent patterns. Edvinsson (2015) suggested using a moving minimum of crude death rates to check for possible underestimation. The search for suspiciously low death rates is based on observations from European population reconstructions that fairly uniformly suggest a pre-industrial mortality floor of about two per thousand. Similar comparative logic underlies Razzell’s (1993) critique of low death rates in Wrigley and Schofield (1981).

Unfortunately, the final output series are often sensitive to the corrections made (e.g., Edvinsson 2015; Lindert 1983:136; Palm 2001; Palma et al. 2017; Razzell 1993), with the result that the various layers of aggregation and error corrections—what Palm (2001:197) calls “number cooking”—can render associating a reliable estimate of error to the end product virtually impossible. To complicate matters further, the correction procedures often yield conservative estimates so that the final output is easily biased to align with the expectations. As Pitkänen and Laakso (1999:17) noted, “one may disregard new information concerning population processes when they are not in agreement with the expectations.” In his critical review of the family reconstitution of England, Steven Ruggles (1999:110) pointed out that the inherent methodological problem of historical population estimates is that we are asked to accept various assumptions as valid when they yield “plausible results,” yet the lack of consensus among historians shows that this “plausibility” is only ever subjective.

## Data

From the eighteenth century onward, Finnish population studies rest on relatively stable ground, and the same applies to Sweden—of which Finland was part, until its annexation to Russia in 1809. The national legislation enacted in 1748 obliged local parishes to compile a population census every three years—the *Tabellverket*—and from 1775 onward, this information was gathered every five years.

When compared internationally, the *Tabellverket* censuses are considered to be of exceptional quality despite some important reservations made concerning the earliest of them (Edvinsson 2015; Pitkänen 1979a; Pitkänen and Laakso 1999). Furthermore, the census form used between 1775 and 1800 permitted double-counting of certain population segments, resulting in the apparent misreporting of population totals during the 1780s and 1790s (Fougstedt and Raivio 1953).^5^

The most pressing issue is that for the first 60 years—that is, until 1815—the *Tabellverket* censuses excluded the southeastern part of Finland, which was annexed to Russia in the peace treaties of 1721 and 1743 and then reannexed to Finland in 1812. The omission is not trivial; the population in these regions totaled some 180,000 people in the 1810s, close to 20% of the total Finnish population. Furthermore, the Greek Orthodox and Roman Catholic population were not included in the population counts until the 1830 census revision, constituting some 2.2% of the total population at the first count. After this, the population data for Finland are of high quality, with incremental improvement over the nineteenth century (e.g., Pitkänen 1993).

In estimating Finland’s population development before and between the high-quality census benchmarks, this study uses parish records of baptisms and burials.^6^ The first statute to stipulate that these records should be kept was issued in 1628, but it was not until 1665 and 1673 that proper judicial measures were taken to enforce the practice and not until the late 1680s that parish registering became relatively common practice (Jutikkala 1944; Muroma 1991; Valpas 1967). A few scattered burial records are available from the 1630s, but 1648 is the first year when both variables are simultaneously available—burials for nine parishes, and baptisms for one. Because of the consequent lack of available registers, vital events before the 1680s have to be estimated from data that are mostly incomplete and provided by fewer than 50 parishes. Subsequently, plenty of prior research has addressed the (un)reliability of registers and the population figures they produce (e.g., Eriksson et al. 2008; Jutikkala 1944, 1965; Kaukiainen 2013; Muroma 1991; Pitkänen 1977a, 1979b; Valpas 1967).

Our parish record data were gathered from HisKi,^7^ the Finnish Genealogical Society’s Internet data bank, which contains the vast majority of Finnish parish records available. HisKi is an ongoing voluntary project to digitize Finnish parish records that started in the 1990s, in which ecclesiastical events are entered into the database one individual at a time. Local studies that have cross-checked the HisKi database against the original records reveal occasional errors, but the digital versions of the registers are overall of high quality (e.g., Uotila 2014).

Using HisKi, we collected a total of 3.8 million entries for baptisms and 2.6 million for burials—every baptism and burial entry available from the early seventeenth century through to 1850. We grouped the 495 parishes available into 197 statistical units. The statistical units were created so that they remain stable over time; for example, none merge or are divided during the period of this study. If a parish remained intact for the duration of the analysis, it was included as it was. In other instances—such as when parishes had been divided—we grouped them to form the original mother parishes.

We used the Finnish borders of 1815, and therefore traced the history of the population of modern Finland.^8^ This accounts for the level shift evident in the *Tabellverket* series, when regions in the southeastern corner that had been annexed to Russia (in 1721 and 1743) were reannexed to Finland in the early nineteenth century.

**Fig. 1:**
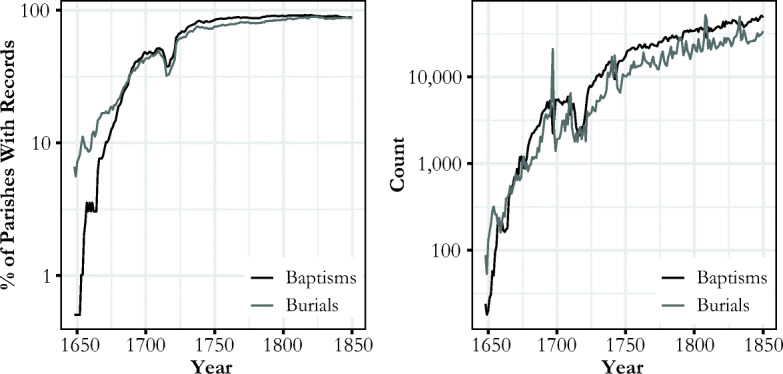
Number of ecclesiastical events in the HisKi database. The total number of statistical units is 197.

Figure 1 presents both the percentage of statistical units with data available and the overall number of events recorded. From this, we can see that except during the Russian occupation in 1713–1721, when many church archives were destroyed, parish records were gradually becoming available with time.

Important omissions in the death registers are military casualties (Edvinsson 2015; Palm 2001; Valpas 1967). To address these omissions, we used the estimates provided in Lappalainen (2001) and Valpas (1967). These are crude figures and are possibly underestimates (Muroma 1991; Palm 2001), but they are in line with the magnitude of losses generally perceived to have occurred during the wars of the seventeenth and eighteenth centuries.

The parish data do not have to be a random sample of all parishes but should be random with respect to the variables of interest (births and deaths). Typical concerns relate to the absence of nonconformists in parish registers, registering deficits during population crises, and the survival/availability of records with respect to the local population growth rate.

The population of Finland before the 1850s was highly homogenous, with nearly all the population belonging to the Lutheran church. We therefore do not think that there is a connection between the missingness of the data and the prevalence of nonconformists.

Although substantial mortality crises could in principle hamper the smooth functioning of parish registers, their overall organization was already reasonably well developed by the late seventeenth century (to the extent that registers were even kept). The famine of the 1690s might be an important caveat to this, and we have duly undertaken special measures to account for the abnormalities that occurred during this mortality surge (described in detail in the following section). Existing contextual literature does not suggest that there would have been mass civilian casualties during the 1700–1721 war, so if there are problems in the quality of the registering during this period, its quantitative impact is most likely minor.^9^

We believe that the overall significance of bias stemming from the sample composition is small with respect to other deficiencies in the data. Importantly, we can compare our final estimates with existing metastudies, such as Pitkänen (2007), that have aggregated local population estimates based on various taxation registers, providing an independent point of comparison.

## Method

With complete parish records, the population process μ would be fully determined by simple accounting as

$$ {\upmu}_t={\upmu}_{t-1}+{B}_t-{D}_t,\kern1em t=1647\dots 1850, $$

where ${B}_t=\sum \limits_i^{197}{b}_{t,i}$ is the total number of births at year *t* for all 197 parishes and similarly for deaths *D*_*t*_. This formulation assumes zero net migration, which we have to resort to, like Galloway (1994) and Edvinsson (2015), because there are no good quality early modern benchmark population estimates that would allow for residual estimation of net migration.

Our observations consisted of registered baptisms *b*_*t*,*i*_ and burials *d*_*t*,*i*_ for the years 1648–1850, compiled from all those parishes that had records for both baptisms and burials for at least 10 years (177 parishes). As described earlier, these records are incomplete in three ways: (1) baptisms did not necessarily represent births, nor burials deaths; (2) for most years, observations came from only some parishes; and (3) some burials and baptisms went unregistered. This incompleteness applied especially to the seventeenth century. In the following, we assumed that the missingness was independent of parish size as well as birth and death rates.

The *Tabellverket* data provide us with censuses of total population for

$$ \mathcal{T}=\left\{1749,1751,1754,\dots, 1772,1775,1780,1800,1805,\dots, 1845,1850\right\}. $$

We did not use the censuses of 1785, 1790, and 1795 for the reasons detailed earlier. Meanwhile, three known deficiencies in the remaining censuses were corrected as follows. First, the missing parishes of southeast Finland before the year 1815 were accounted for by using population censuses conducted in the region in 1754 and 1783 (Kaukiainen 2013). Through a prior assumption that the population in this area grew similarly to the rest of the country, we then interpolated these for the observed census years in $\mathcal{T}$. Second, we corrected the first census of 1749, raising the population by 3.8%, as suggested by Pitkänen (1979a). Third, we accounted for the jump in population in 1830 resulting from the inclusion of the Greek Orthodox and Roman Catholic population in registers beginning in that year. Roughly 2% of the population in 1830 was Greek Orthodox and Roman Catholic (Kilpi 1913, 1915), and so we made a prior assumption that this ratio was similar for earlier periods as well.

**Fig. 2:**
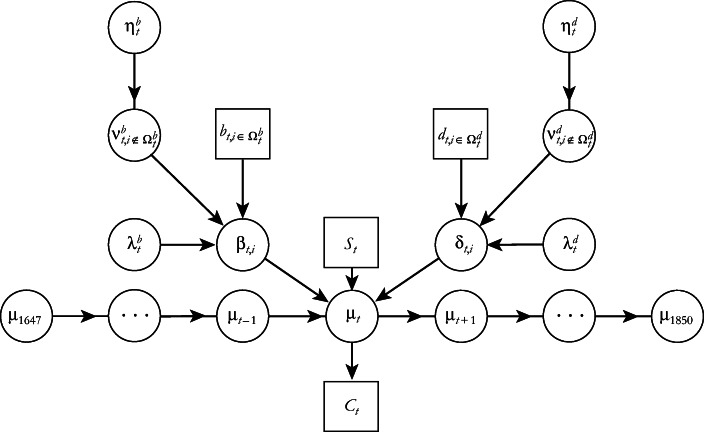
The hierarchical structure of the main components in the population model. The data are shown in boxes, and the unknown variables in circles.

A skeleton of our population model illustrating the hierarchical structure is shown in Fig. 2.^10^ In the following, we define and explain the components of the model, starting from the census data, then moving upward in the figure.

The census years serve as reference points for our model by a simple relation

$$ {C}_t\kern0.5em \sim \kern1em N\left({\upmu}_t,{\upsigma}_c^2\right),\kern1em t\in \mathcal{T}, $$

where μ_*t*_ represents the true (unknown) population size we aim to estimate, and σ_*c*_ is the standard deviation of the measurement noise with a *Gamma* (1,0.0001) prior^11^ translating to a 95% prior interval [253; 36,889]. Because the population increased during the *Tabellverket* era, a constant variance assumption leads to the increasing relative accuracy of census data, consistent with the fact that the first census estimates contain more uncertainty than the latest (e.g., Jutikkala 1944; Pitkänen 1979a; Pitkänen and Laakso 1999).

To provide a prior for the initial population μ_1647_, we used previous literature (Åström 1978; Ignatius 1866; Kilpi 1917; Luukko 1967; Sundquist 1929–1931) to arrive at a mean population estimate of 430,000, with a standard deviation of approximately 30,000.^12^

Because of multiple levels of missingness, we define μ_*t*_ as

$$ {\displaystyle \begin{array}{ll}{\upmu}_{1647}& \sim Gamma\left(215,0.0005\right),\\ {}{\upmu}_t& \sim Gamma\left({\uppsi}_{\upmu}\left({\upmu}_{t-1}+{\upbeta}_t-{\updelta}_t-{s}_t\right),{\uppsi}_{\upmu}\right)\kern0.1em t=1648\dots 1696,1698\dots 1850,\\ {}{\upmu}_{1697}& \sim Gamma\left({\uppsi}_{\upmu}\left({\upmu}_{1696}+{\upbeta}_{1697}-{\upphi}_{\upmu}{\updelta}_{1697}-{s}_{1697}\right),{\uppsi}_{\upmu}\right),\end{array}} $$

where β_*t*_ and δ_*t*_ are birth and death estimates for year *t*, and the deterministic part *s*_*t*_ corresponds to the estimates of military casualties (Edvinsson 2015; Lappalainen 2001; Palm 2001; Valpas 1967), which we took as given. As a prior for ψ_μ_ we used a *Gamma*(2, 4) distribution. The prior for the first year translates to a 95% prior interval of [374,440; 489,348], with a prior mean population of 430,000 and a standard deviation of 29,326.

The extreme effects of the famine in the 1690s were handled separately as follows. Based on earlier literature (e.g., Eriksson et al. 2008; Jutikkala 1944; Muroma 1991), we assumed that in 1695–1697, the population dropped by 15% to 30%. We modeled this event using the ratio π = μ_1697_ / μ_1695_, for which we defined a beta prior with a mean of 0.775 and precision of 200, leading to 99% prior quantiles of [0.695; 0.845]. Based on this, we defined a mortality correction coefficient ϕ_μ_ for the number of deaths in 1697 as

$$ {\upphi}_{\upmu}=\frac{\upmu_{1696}+{\upbeta}_{1697}-{s}_{1697}+\uppi {\upmu}_{1695}}{\updelta_{1697}}, $$

leading to μ_1697_ ∼ *Gamma*(ψ_μ_πμ_1695_, ψ_μ_).

A separate treatment of the 1696/1697 population drop was required because without it, the modeling process would have smoothed out the population drop, creating a spurious decrease in population before the famine, a shallower drop for the famine itself, and a gentler recovery after it. We did not encounter similar problems with respect to other population crises, so we are confident that this kind of treatment is required in only the most extreme cases.

Considering only the previously defined 177 parishes, we denoted those parishes (at year *t*) with recorded baptisms and burials as ${\Omega}_t^b$and ${\Omega}_t^d$, respectively. From this, the birth and death processes were defined as follows:

$$ {\upbeta}_t=\frac{1}{\uplambda_t^b}\left(\sum \limits_{i\in {\Omega}_t^b}{b}_{t,i}+\sum \limits_{i\notin {\Omega}_t^b}\ {\hat{b}}_{t,i}\right), $$

$$ {\updelta}_t=\frac{1}{\uplambda_t^d}\left(\sum \limits_{i\in {\Omega}_t^d}{d}_{t,i}+\sum \limits_{i\notin {\Omega}_t^d}\ {\hat{d}}_{t,i}\right), $$

where the latter terms in parentheses correspond to the parish-level estimates of completely missing baptism and burial records; ${\uplambda}_t^b$ is the proportion of estimated total number of baptisms of all births *B*_*t*_ at year *t*; and ${\uplambda}_t^d$ is defined similarly for burials and deaths.

The λ coefficients took into account the partial missingness in parish records, the omission of 20 parishes, the discrepancy between baptisms and births, and the discrepancy between burials and deaths. As the partial missingness of parish records decreased in time, we defined the λ coefficients as logistic growth curves with fixed endpoints. First, we assumed that the partial missingness in 1850 was equal in both baptism and burial records (${\uplambda}_{1850}^b={\uplambda}_{1850}^d={\uplambda}_{1850}$); denoted *r*_*j*_ and *m*_*j*_ as the growth rate and the midpoint, respectively, of logistic curve for *j* = *b*, *d*; and then defined the standard logistic curve as

$$ {\overset{\sim }{\uplambda}}_t^j=\frac{1}{1+\exp \left(-{r}_j\left(t-{m}_j\right)\right)},j=b,d,\kern1em t=1648\dots 1850, $$

with *Gamma*(2, 20) as prior for *r*_*j*_ and *Beta*(5, 5) as prior for scaled midpoint $\tilde{m}_{j}=\left({m}_j-1647\right)/\left(1830-1647\right)$.

For easier prior incorporation, we then transformed ${\overset{\sim }{\uplambda}}_t^j$ to interval $\left[{\uplambda}_{1648}^{\mathrm{j}},{\lambda}_{1850}\right]$ by scaling

$$ {\uplambda}_t^j=\frac{{\overset{\sim }{\uplambda}}_t^j-{\overset{\sim }{\uplambda}}_{1648}^j}{{\overset{\sim }{\uplambda}}_{1850}^j-{\overset{\sim }{\uplambda}}_{1648}^j}\left({\uplambda}_{1850}-{\uplambda}_{1648}^j\right)+{\uplambda}_{1648}^j,\kern1em j=b,d,\kern1em t=1648\dots 1850. $$

We then defined *Beta*(10*p*/197, 10(1 – *p* / 197)) with *p* = 177 as the prior for λ_1850_, translating to 95% prior interval [0.66; 1]. Instead of defining a prior directly for the first year, we defined priors for proportions ${\uplambda}_{1648}^j/{\uplambda}_{1850}$ with a constraint ${\uplambda}_{1648}^d<{\uplambda}_{1648}^b$; in other words, we assumed that the burial entries were less accurate than those of baptisms. Based on this, we defined ${\uplambda}_{1648}^d/{\uplambda}_{1850}={a}_1$ and ${\uplambda}_{1648}^b/{\uplambda}_{1850}={a}_1+{a}_2$, with *Dirichlet*(10, 5, 5) as a prior for (*a*_1_, *a*_2_, 1 − *a*_1_ − *a*_2_). This led to prior means of 0.5λ_1850_ for ${\uplambda}_{1648}^d$ and 0.75λ_1850_ for ${\uplambda}_{1648}^b$, with 95% prior intervals for the proportions as [0.29; 0.71] and [0.54; 0.91].

Finally, we modeled the parish records as

$$ {b}_{t,i}\sim Gamma\left({\uppsi}_{bd}\exp \left({\nu}_{t,i}^b\right),{\uppsi}_{bd}\right),\kern2.5em i\in {\Omega}_t^b $$

$$ {\hat{b}}_{t,i}\sim Gamma\left({\uppsi}_{bd}\exp \left({\nu}_{t,i}^b\right),{\uppsi}_{bd}\right),\kern2.5em i\notin {\Omega}_t^b $$

$$ {\nu}_{t,i}^b\sim N\left({\nu}_{t-1,i}^b+{\upeta}_t^b,{\upsigma}_{b,\nu}^2\right), $$

$$ {\upeta}_t^b\sim N\left({\upeta}_{t-1}^b,{\upsigma}_{b,\upeta}^2\right), $$

and similarly for burials, except for year 1697, when we added a parish-level correction term ϕ_*d*_ for the famine as

$$ {d}_{t,i}\sim Gamma\left({\uppsi}_{bd}{\upphi}_d\exp \left({\upnu}_{t,i}^d\right),{\uppsi}_{bd}\right),\kern2.5em i\in {\Omega}_t^d $$

$$ {\hat{d}}_{t,i}\sim Gamma\left({\uppsi}_{bd}{\upphi}_d\exp \left({\upnu}_{1697,i}^d\right),{\uppsi}_{bd}\right),\kern1.5em i\notin {\Omega}_t^d $$

$$ \log \left({\upphi}_d\right)\sim N\left(2,{0.25}^2\right). $$

As priors, we used ψ_*bd*_ ~ *Gamma*(2, 4), σ_*j*, *ν*_ ~ *Gamma*(2, 10), and σ_*j*, *η*_ ~ *Gamma*(2, 20), for *j* = *b*, *d*. For prior distributions of year 1648, we used *N*(3.8, 0.5) for ${\upnu}_{1648,i}^b$; *N*(3.2, 0.5) for ${\nu}_{1648,i}^d$; and *N*(0, 0.01) for ${\upeta}_{1648}^j$, for *j* = *b*, *d*, *i* = 1. . . ,177.

We estimated our model using Markov chain Monte Carlo with *rstan* (Stan Development Team 2018), which is an R interface (R Core Team 2019) for the probabilistic programming language Stan (Carpenter et al. 2017). We employed the NUTS sampler (Hoffman and Gelman 2014) with 16 chains that each consisted of 12,500 iterations, with the first 2,500 discarded as a warm-up. With 16 parallel chains, the total computation time was 144 hours.^13^

## Results

Table 1 shows the summary statistics from the posterior draws of time-invariant parameters together with the estimate of Monte Carlo standard error, effective sample sizes, and potential scale reduction factor $\hat{R,}$ which indicate convergence.

**Table 1:** Descriptive posterior statistics for the unknown parameters

	Mean	MCSE	SD	2.5%	25%	50%	75%	97.5%	Effective *N*	$\hat{R}$
σ_*b*, η_	0.15	<0.01	0.01	0.13	0.14	0.15	0.16	0.17	83,601	1.00
σ_*d*, η_	0.30	<0.01	0.02	0.27	0.29	0.30	0.31	0.33	157,076	1.00
σ_*b*, *ν*_	0.09	<0.01	<0.01	0.09	0.09	0.09	0.09	0.09	8,092	1.00
σ_*d*, *ν*_	0.26	<0.01	<0.01	0.25	0.25	0.26	0.26	0.26	14,105	1.00
ψ_*bd*_	0.33	<0.01	<0.01	0.32	0.33	0.33	0.33	0.34	18,522	1.00
ψ_μ_	0.38	0.01	0.29	0.08	0.18	0.29	0.49	1.17	2,353	1.01
σ_*c*_	2,358	21	1,342	177	1,372	2,276	3,187	5,297	3,903	1.00
μ_1647_	440,380	265	29,236	384,595	420,322	439,830	459,869	498,951	12,137	1.00
ϕ_*d*_	5.20	0.01	0.60	4.13	4.78	5.16	5.57	6.46	7,517	1.00
π	0.78	<0.01	0.03	0.72	0.76	0.78	0.80	0.83	125,064	1.00
ϕ_μ_	1.41	<0.01	0.38	0.75	1.15	1.38	1.65	2.24	21,918	1.00
*r*_*b*_	0.07	<0.01	0.06	0.01	0.03	0.05	0.09	0.23	13,416	1.00
*r*_*d*_	0.10	<0.01	0.07	0.01	0.05	0.08	0.13	0.27	22,773	1.00
*m*_*b*_	1,740.16	0.22	23.22	1,695.26	1,724.50	1,739.15	1,755.88	1,785.45	10,881	1.00
*m*_*d*_	1,703.17	0.25	20.88	1,670.44	1,688.66	1,700.29	1,714.23	1,754.17	7,106	1.00
a_1_	0.58	<0.01	0.10	0.37	0.51	0.59	0.66	0.77	19,889	1.00
a_2_	0.18	<0.01	0.07	0.06	0.13	0.17	0.22	0.34	21,053	1.00
a_3_	0.24	<0.01	0.09	0.10	0.18	0.23	0.29	0.43	28,638	1.00
${\uplambda}_{1648}^b$	0.64	<0.01	0.08	0.48	0.59	0.65	0.70	0.79	18,582	1.00
${\uplambda}_{1648}^d$	0.49	<0.01	0.09	0.31	0.43	0.49	0.56	0.67	16,717	1.00
λ_*n*_	0.85	<0.01	0.05	0.76	0.81	0.84	0.88	0.95	7,453	1.00

*Notes:* MCSE is the estimate of the standard deviation of the posterior mean, Effective *N* is the estimate of the effective sample size of the posterior due to the autocorrelation, and $\hat{R}$ is a converge measure that compares the between- and within-chain estimates (should be close to 1.00).

**Fig. 3:**
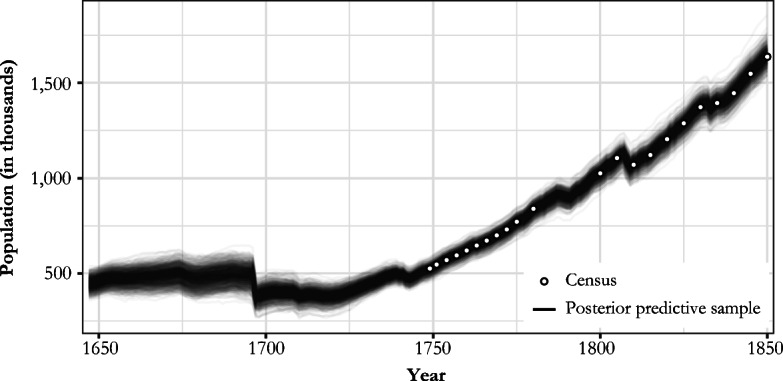
One thousand draws from posterior predictive distribution of population series (lines) and observed population censuses (dots)

The behavior of the model can be conveniently demonstrated with posterior predictive checks. Given the posterior samples of ψ_*bd*_, ${\uplambda}_t^j$, ${\nu}_{t,i}^j$, π, ϕ_δ_, ψ_μ_, μ_1647_, and ${\upsigma}_c^2$, *j* = *b*, *d*, *i* = 1, . . . , 177 and *t* = 1648, . . . , 1850, we simulated new replications of β_*t*_ and δ_*t*_ (assuming empty ${\Omega}_t^j$ for all *t* and *j* = *b*, *d*). Using these, we sampled new latent population processes, μ_*rep*_, and sampled hypothetical census data from *N*(μ_*rep*_, ${\upsigma}_c^2$). Had the model been severely misspecified, these posterior samples would have shown considerably different patterns compared with the real census data; but as shown in Fig. 3, which contains 1,000 replications from posterior predictive distribution, quite the opposite occurred. While the variation in these posterior predictive samples was, as expected, high when compared with our 95% posterior interval (see Fig. 5), the overall trend had a similar shape to census observations.

**Fig. 4:**
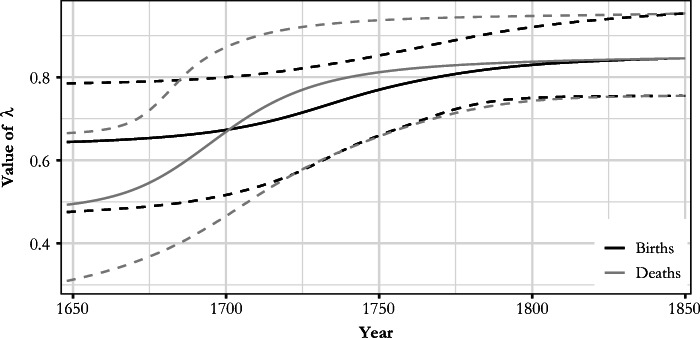
Posterior means and 95% intervals for the λ coefficients

Figure 4 plots the λ coefficients that account for the missingness and various omissions in the parish records. These estimates show that improvement of the parish registers was a long process, as suggested by earlier literature (Pitkänen 1979b; Pitkänen and Laakso 1999). At the beginning of our time span, the quality of entries in HisKi for burials (λ = 0.49) is worse than for baptisms (λ = 0.64). However, burial entries surpass the quality of baptism entries in the early eighteenth century, with both records converging at λ = 0.85 by the early nineteenth century—a value that is close to the proportion of parishes used in the analysis of all parishes (177 / 197 = 0.898).^14^ This implies that data quality issues during the nineteenth century were primarily related to the availability of parish records, rather than the quality of recorded entries (this is visible in Fig. 6).

**Fig. 5:**
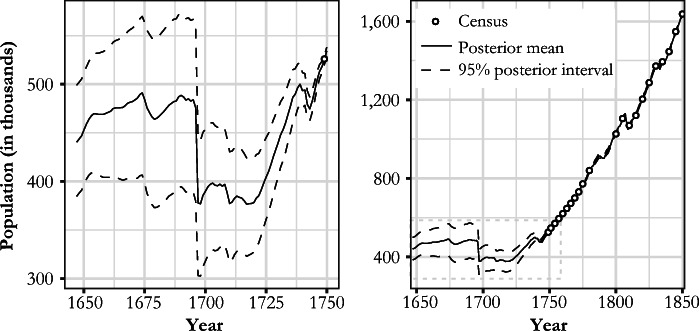
Estimated Finnish population development (1647–1850). Solid lines represent the posterior means, and dashed lines correspond to the limits of the 95% posterior intervals. Dots mark the census points.

**Fig. 6:**
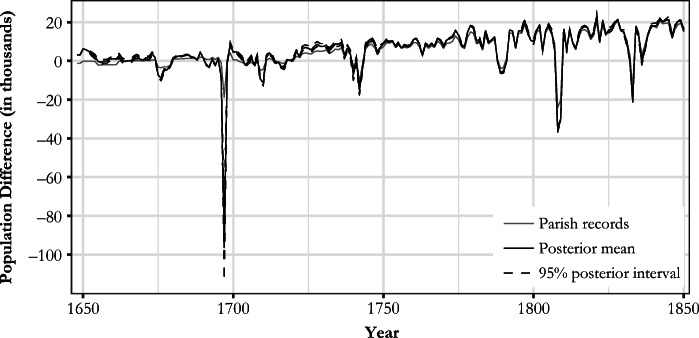
Estimated yearly changes in population levels in comparison to parish records data

Figure 5 shows the estimated Finnish population series for 1647–1748 (the period predating the *Tabellverket*) and the whole estimate (including the census points). The new population estimate with lower (2.5%) and upper (97.5%) uncertainty bounds are tabulated in the online appendix. Our model estimates a population of 440,000 for 1647 with the lower and upper uncertainty bounds at 385,000 and 499,000, respectively. This puts the estimated error band at 26% of the estimated mean. The band remains wide until the 1720s, after which it rapidly narrows to under 10% by 1738 and less than 2% by the time of the first census in 1749. Figure 6 plots the estimated yearly changes in population levels in comparison to changes deduced directly from parish records data and military casualty estimates. This shows the extent to which the model corrects the raw data.

When taking the quality and availability of parish records into account, the uncertainty intervals are reasonable even at the beginning of the estimation period. Furthermore, not all values within the estimated uncertainty intervals are equally likely. To demonstrate this, based on the posterior sample of μ_*t*_, we calculated the probability of various average annual growth rates during the 1647–1690 period. The probability of negative growth (average annual growth <0.0%) was estimated at 11.8%, slow positive growth rate (0.0% to 0.5%) was estimated at 79.6%, and fast growth (>0.5%) was estimated to be 8.7%.

**Fig. 7:**
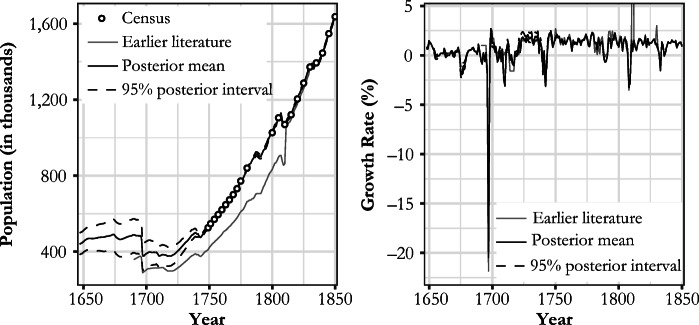
Comparison between the new estimate and previous literature

The new estimate produced in this study and the preexisting Finnish population series are plotted in Fig. 7.^15^ The most obvious feature of this new series is the correction provided to account for the level shifts due to changes in Finnish geographical area. In addition to this, we introduce the following revisions to existing interpretations of Finnish population history. Space constraints allow us to list only the most obvious of these revisions and the areas where further investigation will be required.

1. We produced an estimate of population growth for the latter half of the seventeenth century. The average annual growth rate in 1647–1690 was estimated at 0.24%, with a 95% posterior interval of [–0.22%, 0.61%],^16^ and was lower than what has been suggested or derived from other studies (e.g., Åström 1978; Ignatius 1866; Luukko 1967; Pitkänen 2007; Sundquist 1929–1931). Furthermore, the growth rate was found to be lower than that of the Swedish mainland for the same period (Edvinsson 2015). Our estimate for the eighteenth century differs from Jutikkala’s (1965), especially during the population drop in the 1740s; the estimate for 1780–1787, rather than corroborating the slow growth apparent from the censuses, justifies the criticisms mentioned earlier that have been leveled at the 1785, 1790, and 1795 census totals.
2. According to our estimate, a seventeenth century peak population of 491,000 [406,000, 570,000] occurred in 1674, a level that was not reached until 1738 [1733, 1746].
3. Population was shrinking for multiple years *before* the mortality crisis of 1696–1698. The finding is of importance regarding the wider question of famine causality; the crop failures of 1695 and 1696 did not hit a growing economy. The economic troubles suggested by the pre-famine decline in the 1690s might go a long way to explain the process of cumulative vulnerability that ultimately made the population catastrophe during the winter of 1696/1697 possible.
4. We were better able to quantify the population crises of the 1670s, 1709–1710, and the 1740s than previous research. Although these crises have been previously acknowledged (e.g., Jutikkala 1965; Luukko 1967; Pitkänen 1977b; Valpas 1967; Vilkuna 2005), uncertainty remained about their magnitude.
5. Our estimate of the population decline of 20.6% [14.8%, 26.8%] in 1696/1697 was in line with other studies (e.g., Eriksson et al. 2008; Jutikkala 1944) contradicting the hypothesis that the drop in population was greater (e.g., Muroma 1991). The famine can be justifiably considered as one of the worst in recorded human history (if not *the* worst) in terms of proportional population loss (e.g., Ó Gráda 2009). The overall drop from the highest level of the decade in 1690 to the lowest point of the decade (1698) was 23.2% [17.7%, 29.4%).
6. Our posterior mean for the population in 1721 (383,000, [333,000, 428,000]) was 7,000 less than the estimate for 1700 (390,000, [318,000, 454,000]), when the Great Northern War began. As the population grew during some of the war years, the average maximum drop in population derived from the whole posterior distribution was estimated to be 28,900 [14,900, 57,300], with 1703 and 1717 as the posterior median years for the population peak and trough, respectively. Taken together, our estimate thus favors a “negative” interpretation of the impact of the war, in contrast to those studies suggesting zero growth or even a mild increase in population during the conflict (e.g., Kaukiainen 2013; Strömmer 1969; Valpas 1967).

In addition to the methodological contributions and contributions concerning the early modern Finnish population history, these series provide further evidence of the population recoveries after substantial mortality crises. The population loss induced by the 1690s famine and the 1700–1721 Great Northern War was followed by a growth in population for the rest of the eighteenth century. The Finnish experience of 1695–1721 thus mirrors the population dynamics that occurred after, for example, the Black Death or the Thirty Years’ War in Germany (e.g., Broadberry et al. 2015; Malanima 2003; Pfister and Fertig 2010).

The magnitude of population loss that occurred between 1690 and 1721 changed the trajectory of the Finnish population-economy nexus. After experiencing lower population growth than the Swedish mainland from the 1640s to 1721 (Edvinsson 2015; Palm 2001), Finland became a high-fertility regime upon recovery from war and famine. It took more than 100 years for Finnish and Swedish population growth rates to converge again. The population shock of 1695–1721 might have provided the Finnish economy with an exogenous push toward a high-level equilibrium (Voigtländer and Voth 2013), where the high population growth could have been an outcome of a high resource per capita ratio (Voutilainen 2015).

Finally, as this estimate and existing Swedish population figures show, both Finnish and Swedish populations grew during the latter half of the seventeenth century. The evidence available from both countries also suggests that agricultural production increased and expanded during this period (e.g., Edvinsson 2016; Nummela 2003). Further study is required to determine whether these findings challenge the narrative suggesting that unfavorable climate conditions—also known as the Little Ice Age—seriously hampered economic expansion in the Nordic countries.

## Conclusion

We developed a novel method for estimating population development by applying latent variable Bayesian time-series models to historical parish registers and using existing population censuses as prior information. We demonstrated the applicability of this method by estimating the population levels of Finland starting from the mid-1600s. This marks the first occasion when annual estimates of this kind are available for the whole of Finland (taking the border changes into account), placing the country among the very few with estimates available that date back so far.

We are convinced that our method provides a flexible framework for historical population modeling—one that could be augmented in a number of ways. The details of prior information could be increased, for example, by including prior distributions from birth and death series, as well as more detailed prior standard deviations for cross-sectional population estimates. Further research is needed, however, to assess how the method could be calibrated for optimal use in other contexts.

Our findings suggest that contrary to popular belief, uncertainty intervals for the population estimates are not substantially large. In the Finnish case presented in this article, the wide uncertainty intervals at the beginning were mainly due to data availability and quality concerns. Because these issues are less pressing in other contexts, our results suggest that the statistical properties of population processes themselves help to moderate the errors. We consider this an important lesson and hope it encourages new reconstructions of historical populations and revisiting of existing estimates.

## Electronic supplementary material

ESM 1 (DOCX 32 kb)

## Data Availability

The code and the results are available at https://github.com/helske/finpop.

## Footnotes

1 The most obvious problem arises if the benchmark happens to sit at a bottom of population trough or at the top of a population peak. Interpolation to these kinds of figures can lead to substantially biased long-run growth rates.

2 Any change in population between two periods may result from an infinite number of death, birth, and migration combinations. In practice, because of population structure (e.g., age profile of mortality, distribution of age, sex), the components have finite bounds. This study does not use the so-called projection methods (e.g., Lee 1985; Oeppen 1993; Wrigley and Schofield 1981) that have often been criticized for their strict assumption and input variable requirements (see, e.g., Edvinsson 2015; Palma et al. 2017). An important recent contribution to this projection methodology is provided by Wheldon et al. (2013), who developed a Bayesian model that provides probabilistic estimates of age-specific population counts, fertility rates, mortality rates, and net international migration flows.

3 Wrigley (1997:576–577) drew attention to the generality and contemporaneity of incompleteness of censuses and population statistics.

4 Another variation of this method is to deduct population from household/hearth numbers by multiplying the latter by an assumed mean size (see, e.g., Broadberry et al. 2015:6; Palma et al. 2017).

5 According to censuses, there was a population increase of 15,509 between 1780 and 1785. However, adding the births and subtracting the deaths provided in the vital records contained in the *Tabellverket* gives us a considerably higher figure of 48,236. Between the years 1790 and 1795, the census suggests an increase of 65,208, whereas birth and death registers give a natural increase of 49,954. Censuses, birth, and death records align within an accuracy of 16 people (of 830,000) for the 1795–1800 period. If we consider that these figures represent only net migration, the corresponding rates would be –0.8%, 0.6%, and 0.0004%, respectively. These rates contrast sharply with the average (similarly calculated) net migration rate between 1749 and 1880 of 0.05% (with a standard deviation of 0.0017%). Even during the peak of transatlantic migration in the late nineteenth and early twentieth centuries, net migration dropped to only –0.45% at its lowest. Because there is no credible contextual reason that the disparities in the late 1700s would be due to actual mass-migration events, we see them as stemming from errors in the censuses themselves, as Fougstedt and Raivio (1953) highlighted.

6 Besides the censuses which were collected at regular three- or five-year intervals, the *Tabellverket* data also include annual statistics on births, deaths, and marriages compiled from parish registers. These statistics were produced annually, except in southeast Finland before its reannexation in the 1810s. Parish records, on the other hand, are available throughout the country for the duration of our study. Furthermore, the population change statistics featured in the *Tabellverket* are fairly unique in the international context, so to facilitate the applicability of our method elsewhere, we use parish records to estimate the annual population changes.

7 http://www.genealogia.fi/hiski. The webpage is also available in English.

8 The Paris Peace Treaty of 1947 forced Finland to cede substantial areas to the Soviet Union. The population of these areas, however, left those areas to settle within the borders of modern Finland. This contrasts with treaties from the early modern era, which often saw the population of the occupied areas remaining in the regions that were ceded.

9 It is worth noting that previous estimates point toward stagnation or modest population growth during the war (e.g., Valpas 1967; see also Kaukiainen 2013; Strömmer 1969), whereas the folklore accounts (e.g., Vilkuna 2005) depict outright decades of misery.

10 Our framework bears a resemblance to the Bayesian hierarchical model developed by Wheldon et al. (2013), although the inputs and subsequent outputs differ.

11 We use Gamma distribution with shape and rate parameterization. Compared to Log-Normal distribution, logarithm of Gamma variable is still left-skewed, allowing occasional small values in parish records without overtly affecting the latent processes. Gamma distribution also gives some computational efficiency gains, as the sum of Gamma distributed variables is also Gamma distributed (with same rate parameter).

12 None of these studies used parish registers as a source. Instead, they relied on various fiscal records. This is important because we wanted to avoid using the same set of data twice. We dropped some population estimates that we considered obvious outliers, including extremely low figures in Vaaranen (1860) and excessively high figures presented in Turpeinen (2001). This decision is of little practical importance, however, and in order to be on the safe side, we rounded the estimated standard deviation upward (from c. 28,000) to 30,000.

13 The code and the results are available at https://github.com/helske/finpop.

14 The lambda corrections refer to developments in the HisKi parish register database, rather than parish registers in general. Furthermore, just because there was less need to correct baptisms does not necessarily mean that burials were any less accurately recorded. First, any surviving registers for vital events might have been biased toward births (for reasons unbeknown to us). Second, the genealogical interests of volunteers compiling data for HisKi might have resulted in baptisms being more accurately collected than burials. Furthermore, the lambda correction does not permit us to differentiate the various reasons for omission. The correction coefficient is indifferent as to whether the error stems from underregistering in the first place; from gaps in records caused by, for example, the destruction of church registers; or from nonavailability of some subparish registers altogether.

15 The population series of previous literature is based on the *Tabellverket* censuses and birth and death statistics from 1749 onward. The years 1690–1748 are back-projected from 1749 using vital rates from previous literature: the period 1722–1748 is from Jutikkala (1965); 1698–1721, from Valpas (1967); and 1690–1697, from Muroma (1991).

16 All growth rates and population changes estimated here were derived from the whole posterior distribution, not from only the posterior mean.
