# Supplementary material for: A Bayesian Reconstruction of a Historical Population in Finland, 1647–1850
Source: Demography. 2020 Jun 9;57(3):1171–92. doi: 10.1007/s13524-020-00889-1 (PMC7329763; doi:10.1007/s13524-020-00889-1)
Supplement: Supplementary file 1 — (DOCX 32 kb) [file 13524_2020_889_MOESM1_ESM.docx]

ONLINE APPENDIX.

Table A1. Posterior mean estimates of the population of Finland, 1647-1850, in thousands.

|  | 0 | 1 | 2 | 3 | 4 | 5 | 6 | 7 | 8 | 9 |
| --- | --- | --- | --- | --- | --- | --- | --- | --- | --- | --- |
| 1640 |  |  |  |  |  |  |  | 440 | 444 | 447 |
| 1650 | 453 | 459 | 464 | 467 | 470 | 470 | 469 | 469 | 469 | 470 |
| 1660 | 471 | 472 | 475 | 476 | 475 | 476 | 476 | 478 | 480 | 481 |
| 1670 | 482 | 484 | 486 | 489 | 491 | 484 | 476 | 471 | 466 | 464 |
| 1680 | 465 | 467 | 470 | 472 | 475 | 478 | 480 | 482 | 483 | 487 |
| 1690 | 489 | 487 | 484 | 485 | 483 | 484 | 476 | 378 | 377 | 385 |
| 1700 | 390 | 394 | 397 | 398 | 396 | 395 | 397 | 395 | 397 | 388 |
| 1710 | 377 | 379 | 383 | 385 | 384 | 383 | 380 | 376 | 377 | 377 |
| 1720 | 378 | 383 | 385 | 391 | 398 | 404 | 410 | 417 | 425 | 433 |
| 1730 | 440 | 448 | 453 | 460 | 469 | 477 | 486 | 490 | 496 | 500 |
| 1740 | 493 | 494 | 479 | 475 | 482 | 493 | 504 | 513 | 518 | 525 |
| 1750 | 534 | 545 | 555 | 565 | 572 | 582 | 588 | 596 | 603 | 612 |
| 1760 | 623 | 634 | 643 | 646 | 655 | 663 | 671 | 679 | 691 | 701 |
| 1770 | 709 | 720 | 731 | 743 | 759 | 772 | 781 | 788 | 803 | 821 |
| 1780 | 839 | 849 | 860 | 866 | 881 | 891 | 902 | 914 | 914 | 907 |
| 1790 | 902 | 901 | 917 | 933 | 939 | 955 | 970 | 991 | 1007 | 1017 |
| 1800 | 1027 | 1043 | 1062 | 1067 | 1083 | 1102 | 1116 | 1122 | 1087 | 1056 |
| 1810 | 1071 | 1077 | 1094 | 1103 | 1109 | 1122 | 1140 | 1158 | 1175 | 1188 |
| 1820 | 1203 | 1226 | 1235 | 1256 | 1270 | 1286 | 1302 | 1322 | 1343 | 1359 |
| 1830 | 1373 | 1383 | 1384 | 1362 | 1380 | 1393 | 1392 | 1397 | 1410 | 1428 |
| 1840 | 1446 | 1464 | 1487 | 1507 | 1527 | 1549 | 1561 | 1579 | 1599 | 1621 |
| 1850 | 1636 |  |  |  |  |  |  |  |  |  |

For the years after 1850, the Finnish population figures are available through Statistics Finland:

http://pxnet2.stat.fi/PXWeb/pxweb/en/StatFin/StatFin__vrm__kuol/statfin_kuol_pxt_12at.px/

Table A2. Lower limit (2.5 %) of the estimated 95 % posterior interval of the population size, 1647-1850, in thousands.

|  | 0 | 1 | 2 | 3 | 4 | 5 | 6 | 7 | 8 | 9 |
| --- | --- | --- | --- | --- | --- | --- | --- | --- | --- | --- |
| 1640 |  |  |  |  |  |  |  | 385 | 388 | 391 |
| 1650 | 397 | 402 | 406 | 409 | 410 | 408 | 406 | 405 | 404 | 404 |
| 1660 | 404 | 404 | 406 | 405 | 403 | 402 | 401 | 402 | 403 | 403 |
| 1670 | 402 | 403 | 403 | 406 | 406 | 398 | 388 | 382 | 376 | 373 |
| 1680 | 373 | 375 | 377 | 379 | 382 | 385 | 386 | 388 | 388 | 393 |
| 1690 | 395 | 392 | 389 | 390 | 388 | 389 | 380 | 303 | 303 | 312 |
| 1700 | 318 | 324 | 327 | 330 | 329 | 329 | 332 | 331 | 335 | 327 |
| 1710 | 316 | 319 | 325 | 328 | 327 | 328 | 326 | 323 | 324 | 325 |
| 1720 | 327 | 333 | 337 | 344 | 353 | 361 | 368 | 377 | 387 | 396 |
| 1730 | 405 | 415 | 421 | 430 | 440 | 450 | 461 | 466 | 474 | 480 |
| 1740 | 476 | 478 | 465 | 462 | 471 | 483 | 495 | 505 | 512 | 520 |
| 1750 | 529 | 540 | 551 | 561 | 569 | 577 | 584 | 593 | 599 | 608 |
| 1760 | 619 | 630 | 639 | 642 | 651 | 658 | 667 | 675 | 687 | 698 |
| 1770 | 705 | 715 | 727 | 739 | 755 | 768 | 776 | 783 | 797 | 816 |
| 1780 | 833 | 843 | 853 | 858 | 873 | 881 | 892 | 903 | 903 | 896 |
| 1790 | 890 | 889 | 905 | 921 | 928 | 944 | 961 | 983 | 999 | 1011 |
| 1800 | 1023 | 1038 | 1055 | 1060 | 1076 | 1095 | 1109 | 1114 | 1080 | 1049 |
| 1810 | 1067 | 1071 | 1087 | 1097 | 1103 | 1118 | 1134 | 1152 | 1169 | 1182 |
| 1820 | 1198 | 1219 | 1229 | 1249 | 1264 | 1281 | 1296 | 1315 | 1336 | 1353 |
| 1830 | 1369 | 1377 | 1377 | 1355 | 1374 | 1388 | 1386 | 1390 | 1403 | 1422 |
| 1840 | 1441 | 1458 | 1480 | 1500 | 1521 | 1544 | 1555 | 1572 | 1592 | 1614 |
| 1850 | 1631 |  |  |  |  |  |  |  |  |  |

Table A3. Upper limit (97.5 %) of the estimated 95 % posterior interval of the population size, 1647-1850, in thousands.

|  | 0 | 1 | 2 | 3 | 4 | 5 | 6 | 7 | 8 | 9 |
| --- | --- | --- | --- | --- | --- | --- | --- | --- | --- | --- |
| 1640 |  |  |  |  |  |  |  | 499 | 502 | 505 |
| 1650 | 512 | 518 | 523 | 528 | 531 | 532 | 532 | 533 | 534 | 535 |
| 1660 | 538 | 539 | 543 | 545 | 546 | 547 | 548 | 551 | 554 | 556 |
| 1670 | 558 | 560 | 563 | 567 | 570 | 563 | 555 | 551 | 547 | 546 |
| 1680 | 547 | 550 | 553 | 556 | 559 | 563 | 565 | 567 | 567 | 572 |
| 1690 | 573 | 571 | 567 | 568 | 566 | 567 | 558 | 444 | 442 | 450 |
| 1700 | 454 | 458 | 460 | 460 | 457 | 456 | 456 | 453 | 454 | 443 |
| 1710 | 430 | 431 | 434 | 436 | 434 | 432 | 429 | 424 | 424 | 423 |
| 1720 | 424 | 428 | 429 | 434 | 440 | 445 | 449 | 456 | 463 | 469 |
| 1730 | 475 | 482 | 486 | 492 | 499 | 507 | 515 | 516 | 521 | 522 |
| 1740 | 512 | 511 | 493 | 487 | 493 | 502 | 513 | 520 | 523 | 529 |
| 1750 | 538 | 548 | 559 | 569 | 576 | 586 | 593 | 600 | 608 | 617 |
| 1760 | 627 | 639 | 647 | 649 | 659 | 667 | 675 | 683 | 695 | 705 |
| 1770 | 713 | 724 | 734 | 747 | 763 | 776 | 785 | 793 | 808 | 826 |
| 1780 | 843 | 855 | 867 | 874 | 890 | 900 | 912 | 925 | 925 | 919 |
| 1790 | 914 | 914 | 929 | 944 | 951 | 965 | 980 | 1000 | 1015 | 1024 |
| 1800 | 1032 | 1049 | 1068 | 1073 | 1089 | 1106 | 1123 | 1130 | 1093 | 1063 |
| 1810 | 1078 | 1084 | 1101 | 1110 | 1116 | 1128 | 1146 | 1164 | 1181 | 1193 |
| 1820 | 1207 | 1231 | 1242 | 1263 | 1276 | 1290 | 1308 | 1328 | 1350 | 1365 |
| 1830 | 1379 | 1389 | 1390 | 1370 | 1386 | 1397 | 1398 | 1404 | 1417 | 1434 |
| 1840 | 1451 | 1471 | 1493 | 1514 | 1534 | 1554 | 1568 | 1586 | 1607 | 1627 |
| 1850 | 1641 |  |  |  |  |  |  |  |  |  |
